# Motions around conserved helical weak spots facilitate GPCR activation

**DOI:** 10.1002/prot.26179

**Published:** 2021-07-26

**Authors:** Janne M. Bibbe, Gert Vriend

**Affiliations:** ^1^ CMBI, Radboudumc Nijmegen Netherlands

**Keywords:** activation, GPCR, helix weak spots, mobility, sodium release

## Abstract

G protein‐coupled receptors (GPCRs) participate in most physiological processes and are important drug targets in many therapeutic areas. Recently, many GPCR X‐ray structures became available, facilitating detailed studies of their sequence‐structure‐mobility‐function relations. We show that the functional role of many conserved GPCR sequence motifs is to create weak spots in the transmembrane helices that provide the structural plasticity necessary for ligand binding and signaling. Different receptor families use different conserved sequence motifs to obtain similar helix irregularities that allow for the same motions upon GPCR activation. These conserved motions come together to facilitate the timely release of the conserved sodium ion to the cytosol. Most GPCR crystal structures could be determined only after stabilization of the transmembrane helices by mutations that remove weak spots. These mutations often lead to diminished binding of agonists, but not antagonists, which logically agrees with the fact that large helix rearrangements occur only upon agonist binding. Upon activation, six of the seven TM helices in GPCRs undergo helix motions and/or deformations facilitated by weak spots in these helices. The location of these weak spots is much more conserved than the sequence motifs that cause them. Knowledge about these weak spots helps understand the activation process of GPCRs and thus helps design medicines.

## 1 INTRODUCTION

G protein‐coupled receptors (GPCRs) are responsible for the majority of cellular responses to hormones and neurotransmitters as well as the senses of sight, smell, and taste.^1^ They form the largest family of membrane receptors^2^ and signal through nucleotide exchange on heterotrimeric G proteins. GPCRs are the most important drug target family for the pharmaceutical industry.^3^

The X‐ray structure determination of the first GPCR (rhodopsin,^4^ PDB ID 1F88) revealed the expected seven transmembrane helices, but it also revealed many surprises, such as the presence of the cytosolic helix 8, the location of the loop between transmembrane helices (TMs) 4 and 5, and a large number of helix irregularities such as π‐helix bulges and 3_10_ helix constrictions. GPCRs are—certainly in vitro—rather unstable molecules, which makes it hard to crystallize them. GPCR structures have been solved after stabilization by, for example, protein fusions, the use of exotic cocktails of lipids and metal ions, or by binding ligands, nanobodies, antibodies, or other peptides, normally combined with several mutations.^5^ The resulting wealth of structural data has been used for many purposes, including improved sequence alignments (www.gpcrdb.org ^6^, ^7^). In this article, we concentrate on class A GPCRs, and we strictly follow the GPCRdb generic residue numbering system that deals with all helix irregularities.^7^

**FIGURE 1:**
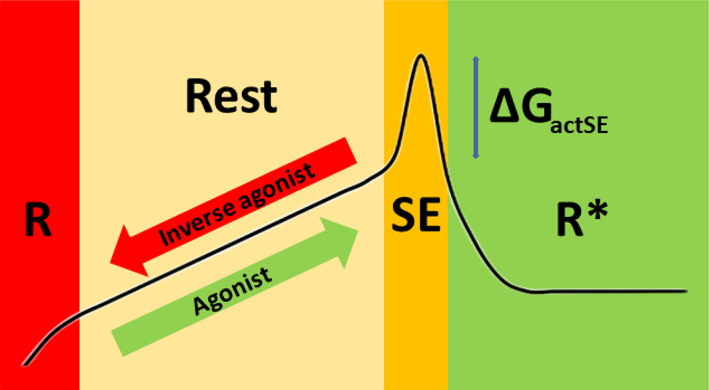
State diagram for GPCR activation. Colored areas represent ensembles of states. The black curve indicates the Δ*G* of all structures with that state. Normal thermal fluctuations can move inactive receptors from left to right in the R‐Rest area and will very occasionally flip the receptor over the Δ*G*_actSE_ barrier. Agonists will bind to a Rest state close to SE and inverse agonists will bind to a Rest state close to or in R. An agonist will move the state of the receptor closer to this Δ*G*_actSE_ barrier, which means that the receptor will be more likely to flip into the R* state due to thermal fluctuations. In the R* state the sodium ion has escaped to the cytosol. The sodium concentration in the cytosol typically is 10 times lower than outside the cell, which aids to the fact that the activation process is nearly irreversible. Borders between colored areas and relative heights of their Δ*G* are arbitrary. The Δ*G* between the bottom of the SE barrier and R* is on the order of 1 kcal/Mole because the sodium gradient over the membrane typically is a factor 10

Figure 1 shows the GPCR activation path from the R and Rest state via the so‐called sodium‐escape (SE) state to the R* state mapped on a state diagram. Agonists will bind to a state that is close to SE or, in other words, move the state of the receptor towards SE. Inverse agonists will bind to a receptor in state R or, in other words, move the state of the receptor towards R. We do not know what the structures in the four states look like and Figure 1 merely shows what might happen in terms of Δ*G*.

The important functional role of the sodium ion that seems bound in GPCRs between the ligand binding cavity and the cytosolic side of the helix bundle is becoming apparent.^8^ This sodium is prevented from escaping to the cytosol by a ring of three aliphatic residues that includes the very conserved Leu 2x46. Only a minor rotameric reorientation of this leucine is required to allow the sodium to move to the cytosol.^9^ This cytosolic escape of sodium critically contributes to the activation process; the low cytosolic sodium concentration makes that the path back from R* to Rest is energetically very unfavorable.

**FIGURE 2:**
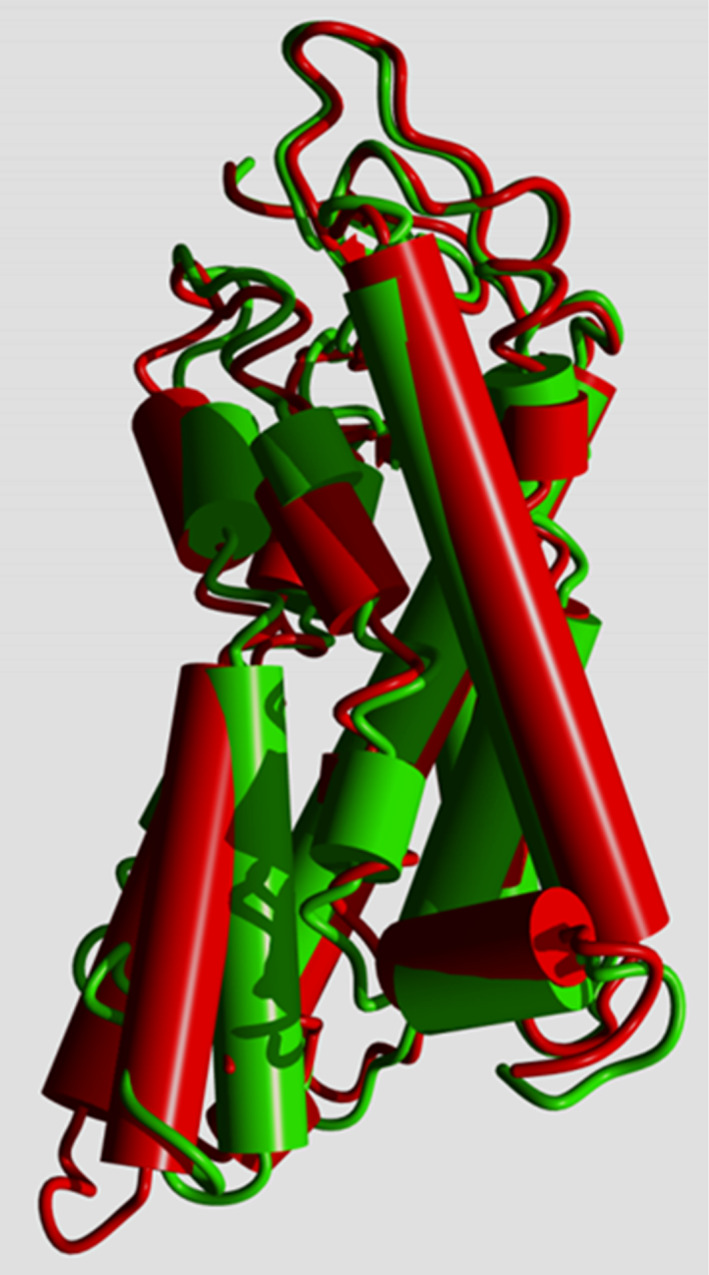
Inactive (green; PDB ID 1U19^10^) and active (red; PDB ID 4J4Q^11^) rhodopsin. Rhodopsin was activated by replacing retinal by octylglucoside. Activation results in opening the cytosolic side (bottom) of the helix bundle. Examples of GPCR structures in the PDB that show a similar opening of the bundle are the adenosine receptor (PDB ID 5G53^12^) and the β2‐adrenoceptor (PDB IDs 4LDE, 4QKX, and 3P0G^13^, ^14^, ^15^). In the adenosine receptor the active state was stabilized by an engineered G protein while the three β2‐adrenoceptor structures were stabilized with nanobodies. Rhodopsin structures that were activated by adding fragments of G proteins also showed similar structural rearrangements

In R* the cytosolic side of the helix bundle opens (Figure 2) so that the G protein can bind “between” the helices. In this process, GPCRs undergo again a large series of rearrangements in their transmembrane helices.^5^

Different GPCRs use different combinations of residues to allow the same motions to take place, and in this article, we will show that these small motions that facilitate the activation path in Figure 1 are very conserved among class A GPCRs.

The main consequence of the model illustrated in Figure 1 is that mutations that stabilize TM helices are likely to reduce the binding of agonists and will make it more difficult for a receptor to spontaneously reach the SE state. The binding of antagonists, on the other hand, is much less likely influenced by these same mutations. The model can explain how allosteric modulators might work. It is likely—but not strictly needed for the validity of the model—that stabilizing mutations increase the binding affinity of inverse agonists.

## 2 MATERIALS AND METHODS

GPCR structures were obtained from the PDB.^16^, ^17^ The list of GPCR structures used is available from the Supporting Information. Sequence alignments were obtained from the GPCRdb,^6^, ^7^ sequence logos were created from an alignment of all Class A GPCRs using WebLogo^18^, ^19^ and conserved sequence motifs were extracted by visual inspection of the alignments and logos. Interactive structure visualization and all molecular manipulations were done with YASARA.^20^ Figures 6 and 7 were created using PyMol.^21^

Structure superposition was done with the WHAT IF^22^ superposition module^23^ as implemented in YASARA.^20^ Hydrogen bond calculations were performed with the WHAT IF hydrogen bond module as implemented in YASARA. Cavities and caves were calculated using the method by Voorintholt et al^24^ (as implemented in the YASARA‐Twinset software) with a probe of 1.4 Å radius. This method places a 1 Å spaced grid over the molecule and puts a positive value at all grid points that are more than 1.4 Å away from any atom. Visualization is then performed using the same contouring software as used by crystallographers to contour electron density. Rotamer distributions were calculated using the method of Chinea et al.^25^ In short, the rotamer distribution software searches in the PDB for stretches of five residues that have a very similar backbone as observed in the local structure, and that have the same middle residue as the pentamer in the local structure. The pentamers so obtained will be superposed on the local structure, but only the side chain of the middle residue is shown.

Information about the activity of mutated GPCRs was obtained from the GPCRdb.^6^, ^26^

## 3 RESULTS AND DISCUSSION

### 3.1 Helix‐weakening residues facilitate activation‐associated motions

Most GPCR helices contain Pro, Gly, Asp, Asn, Ser, and Thr (PGDNST) residues that one would not expect abundantly present in a regular α‐helix. Proline weakens the helix because the backbone nitrogen atom does not have a proton so that it always lacks one hydrogen bond. Glycine has a very flexible backbone that can facilitate local deformations by accepting φ,ψ torsion angles that are energetically unfavorable for the other residue types. Asparagine, aspartic acid, serine, and threonine have small side chains that do not lose much rotameric entropy when a hydrogen bond is formed with the local backbone. When such a hydrogen bond is formed, the backbone and side chain exert a force on each other that leads to backbone atom displacements away from the ideal positions observed in regular α‐helices. PGDNST, especially proline, have often been associated with functionally important kinks in GPCR helices^27^, ^28^ and they are found abundantly in the aforementioned conserved sequence motifs.^29^, ^30^ The helix irregularities that are observed near the PGDNST residues are weak spots that facilitate local rearrangements required to move between states in Figure 1. These weak spots are observed in all helices but TM3. TM3 does not undergo any large structural reorganization upon GPCR activation as was shown by Van der Kant et al who compared many inactive and activated GPCR structures in real space and in distance space.^5^ Consequently, the sequence motif that characterizes TM3 (C,X_23_,DRY) is not involved in helix weakening.

Our finding that different GPCRs use different combinations of residues to allow for the same molecular rearrangements upon activation can only be understood in the context of the four‐state model depicted in Figure 1. We will therefore first review the evidence in support of this model.

### 3.2 Stabilizing mutations influence agonist binding but not antagonist binding

The GPCRdb mutation collection^6^, ^26^ holds information about the activity of many mutated GPCRs. The examples confirm that removing helix‐weakening residues by mutation decreases agonist binding while not influencing antagonist binding. Some examples are shown in Table 1.

**TABLE 1:** Examples of the influence of stabilizing mutations on ligand binding

Mutation	Receptor	Agonist effect	Other effects
S^5x43^A	5‐HT_1A_	Serotonin affinity 95‐fold decreased	Pindolol not affected
S^5x44^A	5‐HT_2A_	Decrease in affinity for serotonin and methylated serotonin	Binding to antagonists not affected (ketanserin, ritanserin, spiperone)
P^5x50^A	M3	Affinity for acetylcholine and carbachol decreased	NMS and 4‐DAMP not affected
C^6x47^T	β_2_	Constitutive activity	
C^7x41^A	M_4_	Lower affinity for acetylcholine	No significant effect on allosteric modulators

*Note*: The GPCRdb lists the mutations used to investigate GPCR activity and affinity for agonists, inverse agonists, and antagonists. This table contains examples of mutations that remove or introduce helix‐weakening residues.

The S^5x43^A mutation in the 5‐HT_1A_ receptor^31^ results in a 95‐fold decrease in the affinity for the endogenous agonist serotonin, but binding of the antagonist pindolol is not affected. The activity of this mutant is lower than that of the wild type.^32^ The S^5x44^A mutation in the 5‐HT_2A_ receptor also results in a decrease in affinity for serotonin and other agonists, while, again, binding to antagonists is not affected. The activity of this mutant does not decrease significantly.^31^ The P^5x50^A mutation in the M_3_ receptor^33^ resulted in decreased affinity for the agonists acetylcholine and carbachol, while the affinity for the antagonists NMS and 4‐DAMP remained unchanged. The activity of the mutated receptor is slightly reduced.^33^ The cysteine at position 6x47 in the β_2_‐adrenoceptor has been mutated to threonine,^34^ which resulted in a constitutively active receptor.^34^ The cysteine can form a weak hydrogen bond that destabilizes the helix (see Figure 6—TM6). When the cysteine is mutated to threonine, the hydrogen bond will pull the helix backbone out of place more strongly. Most aminergic GPCRs have a glycine at position 7x41, but the muscarinic acetylcholine receptors have a cysteine at that position, which destabilizes the helix because it is pushed into the helix by Trp 6x48. A C^7x41^A mutation in the M_1_ receptor^35^ causes a lower affinity for acetylcholine, but no effects can be found on antagonist binding.^35^

The GPCRdb lists an absence of significant effects on ligand binding for the mutations X^4x53^S in several receptors (5‐HT_6_ receptor, MC_2_ receptor, TSH receptor, rhodopsin, medium‐wave sensitive opsin), and C^7x45^A and C^7x45^N in the 5‐HT2C receptor. The C^7x41^S mutation in the M_1_ receptor results in a lower affinity for its agonist carbachol and a higher affinity for its antagonist atropine,^36^ which is opposite to our expectations. Visual inspection suggests that the serine at position 7x41 occupies another rotamer than the cysteine and actually seems to make the helix more stable. The C^6x47^A mutation in the α_1B_‐adrenoceptor results in an increased agonist affinity,^37^ which is hard to explain because alanine generally stabilizes helices. However, we do observe that Cys 6x47 can form a weak hydrogen bond with Asn 7x45 that in turn points with its side chain oxygen into the sodium pocket probably leading to diminished communication between agonist binding and the sodium binding site. The P^4x59^A mutations in the M_1_ ^38^ and M_3_ ^33^ receptor result, as expected, in a large decrease in activity, but the affinity of these receptors for both agonists and antagonists is reduced.^33^, ^38^ It seems that the change in structure of the receptor caused by P^4x59^A is so large that the affinity for all ligands is diminished.

### 3.3 Mutations to support crystallization reduce helix flexibility

Crystallographers want a homogeneous GPCR sample for crystallization attempts. They want all GPCRs in their crystallization experiment to be in the same state, and thus must stabilize that one particular state relative to all other states. To obtain a homogeneous sample they often introduce mutations that reduce potential helix mobility. These mutations tend to stabilize the R and Rest states and thus are expected to reduce the binding affinity of agonists (as agonists bind to a state near SE). Antagonist binding is not associated with the same small (activating) motions and therefore stabilizing mutations—that reduce these motions—are not expected to influence antagonist binding. Many combinations of stabilizing mutations can be observed in PDB files for GPCR structures and many mutations can be observed in more than one receptor. Table 2 lists about 30 examples.

**TABLE 2:** Mutations made to support GPCR crystallization

Helix 1	Helix 2	Helix 3	Helix 4	Helix 5	Helix 6	Helix 7	Other
A^1x54^L R^1x59^S C^1X60^Y	L^2x40^A A^2x52^L M^2x53^V L^2x46^A T^2x62^A	F^3x34^A L^3x41^W M^3x41^W G^3x49^A E^3x49^A R^3x55^A Q^3x37^A	K^4x43^A G^4x60^N	Y^5x58^A Y^5x58^F L^5x63^A	A^6x27^L A^6x33^D T^6x36^A L^6X37^A V^6x41^A	F^7x37^A S^7x41^A F^7x42^A V^7x44^A F^7x48^M	G^215^A K^8x49^E

*Note*: The GPCRdb lists the mutations used to crystallize GPCRs. The examples listed in this table are selected because of the mechanisms through which they stabilize the receptors.

The 5‐HT_1B_ and 5‐HT_2B_ receptor structures always have the mutation L^3x41^W or M^3x41^W, respectively. This X^3x41^W mutation is also found in four out of 20 β2‐adrenoceptor structures, one of the 18 β1‐adrenoceptor structures, all of the CXCR4 structures and the D3 receptor structure. This mutation was first used in a β2‐adrenoceptor structure. The rationale for the introduction of this mutation was that tryptophan was highly conserved at 3x41 in rhodopsin where it contacts both TM4 and TM5, and thus influences the dynamics of the receptor profoundly. The β2‐adrenoceptor is in vitro much less stable than rhodopsin and this tryptophan residue has been suggested to be one of the reasons.^39^ The tryptophan is located near the weak spot in TM5 that is caused by Pro 5x50. The side chain of the tryptophan can interact with the unpaired backbone carbonyl group of the residue at position 5x46. In this way, it decreases the instability caused by Pro 5x50.^39^ Three of the CXCR4 structures also contain a T^6x36^P mutation. This mutation ensures that the helix ends at this position, thus inhibiting any length change of the helix in the activation process. A T^3x36^A mutation is often introduced in the A2A receptor to ease crystallization. This threonine forms a hydrogen bond with the local backbone that destabilizes the helix. Mutating it is therefore likely to stabilize the receptor. Together with A^2x52^L, R^3x55^A, K^4x43^A, L^5x63^A, L^6x37^A, V^6x41^A, and S^7x41^A, this mutation gives the receptor a higher affinity for the inverse agonists ZM241385, XAC, and for caffeine.^40^ Threonine 3x36 and serine 7x41 are located at the bottom of the agonist binding pocket, and reverting these mutations increases activity but does not increase agonist affinity.^40^ This means that they influence the stability (and thus activity) of the receptor in another way.^40^ The A2A structure with the thermostabilizing mutations L^2x46^A, Q^3x37^A, A^2x52^L, and T^2x62^A is said to be in an intermediate state between active and inactive.^41^ The leucine and glutamine are involved in intra‐helical interactions that stabilize the active state, but the alanine and threonine are not known to be involved,^41^ and we hypothesised^9^ that the leucine is critically involved in preventing cytosolic escape from the sodium in the inactive state, which might be the main explanation for the in‐between activation state of the mutated A2A receptor. The combined mutations R^1x59^S, M^2x53^V, Y^5x58^A, A^6x27^L, F^7x37^A, and F^7x48^M stabilize the structure of the β1‐adrenoceptor with a bound antagonist.^42^, ^43^ The mutated receptor has an unaltered affinity for its antagonists, but binds its agonists 2‐3 orders of magnitude less well.^43^ Combining thermostabilizing mutations that are near to each other in the sequence does not greatly improve thermostability,^43^ which makes sense because one mutation should normally be enough to abolish a helix weak spot. The structure of the CCR5 receptor contains four mutations.^44^ Clearly the G^4x60^N improves antagonist binding, the A^6x33^D stabilizes the environment of the DRY motif in TM3, and the C^1x60^Y stabilizes the TM7 ‐ helix 8 corner through several favorable interactions. Neither the structure, nor the associated article^44^ give any hint why K^8x49^E stabilizes the receptor. We lack information to draw strong conclusions, but it might well be that the three mutations, leaving out K^8x49^E, would have worked even better than the combination of four mutations used. L^2x40^A, F^3x34^A, G^3x49^A and Y^5x58^F increase thermostability and expression of the FFA1 receptor.^45^ The receptor had the same affinity for the partial agonist TAK‐875 but the activity was a thousand times lower, probably because the mutations restrained the conformational changes that are needed for activity.^45^ Additionally, the tyrosine at position 5x58 plays an important role in the transmission of signal to the G protein. We studied eight NTS1 receptor structures that together contain 35 different mutations. Four of the structures contain 11 point mutations to increase the expression of the receptor in *E*. *coli*.^46^ Expression of wild‐type GPCRs in prokaryotic cells generally is very low^46^ and these mutations are one of the steps taken to increase the production yield. The first structure of the NTS1 receptor contained six thermostabilizing mutations (A^1x54^L, E^3x49^A, L^6x37^A, F^7x42^A, and V^7x44^A and a glycine to alanine mutation in ECL2) that disabled G protein activation.^47^ When the E^3x49^A, L^6x37^A, and F^7x42^A mutations were reverted, the receptor was able to catalyze nucleotide exchange at the G protein,^48^, ^49^ which is not surprising given the importance of these residues for G‐protein coupling.

### 3.4 Allosteric effects

**FIGURE 3:**
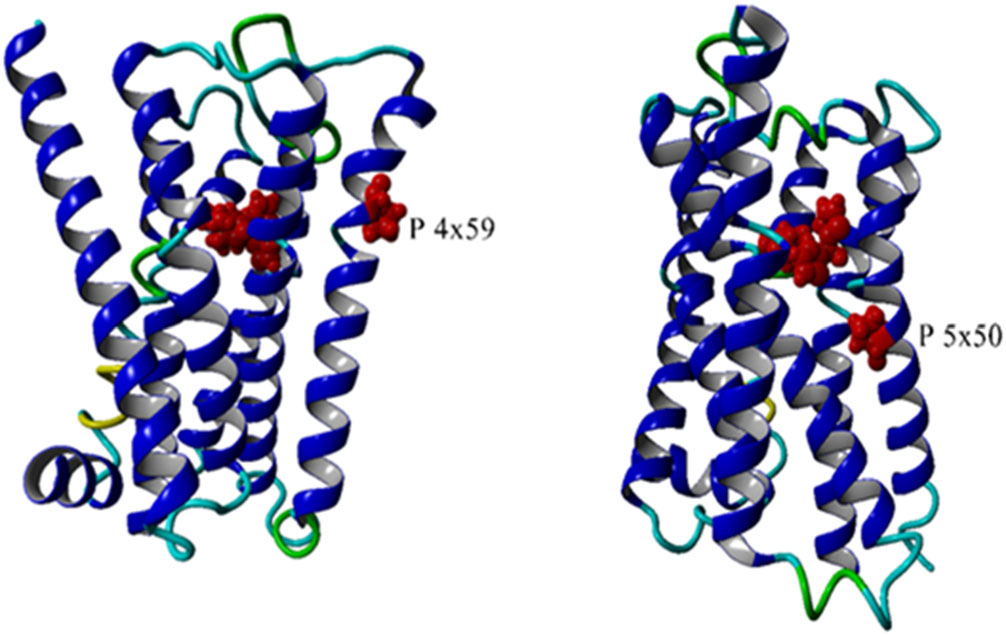
Allosteric mutations. The location is shown of two proline to alanine mutations that lower activity and agonist affinity of the M_3_ receptor.^33^, ^38^ The structure is the un‐mutated M_3_ receptor 4UI5.^50^ The two prolines are shown as red sphere models and have their residue numbers indicated. The large red molecule is an antagonist bound in the main ligand binding pocket

Mutations can influence the binding of all types of ligands by modifying the mobility that is needed to reach the state to which they can bind. Consequently, ligand binding can be influenced by mutating residues that do not have a direct atomic contact with that ligand. Many such allosteric mutations are known. Figure 3 shows two examples of mutations that must exert their effect allosterically through influencing receptor mobility.

This same reasoning can explain how the lipid composition and the cholesterol concentration can influence the apparent binding constant of ligands.

### 3.5 Small motions facilitated by helix weak spots culminate in sodium escape

**FIGURE 4:**
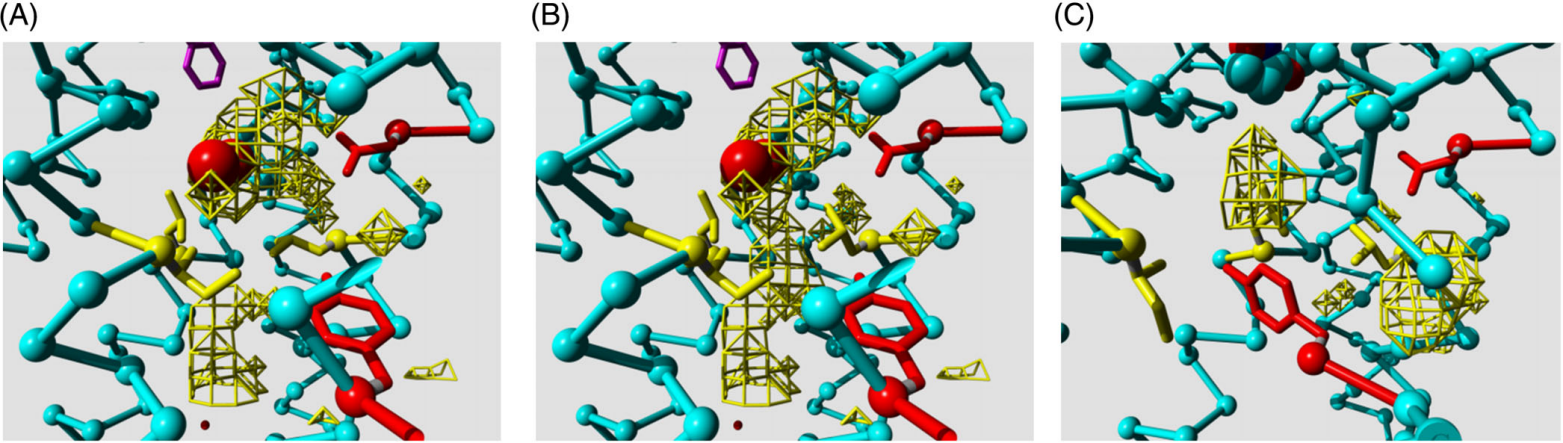
The sodium escape channel. The local environment of the sodium site in respectively. (A) 3UON^52^, ^53^; (B) 3UON with Leu 2x46 modeled in the open rotamer; (C) the agonist‐activated state without sodium in 4MQT.^53^, ^54^ C‐alpha traces are shown in light‐blue (some residues in the front that obscured the view have been removed from the plots for clarity). Two water molecules observed in 3UON near the presumed sodium location are shown as red balls. Cavities large enough to hold water molecules are represented by yellow chicken wire. The cavity below the sodium site extends to the cytosol. A small part of the ligand in 3UON is seen in purple in panels (A) and (B). The side chains of the three aliphatic residues Leu 2x46, Leu 3x43, and Ile 6x40, are shown as yellow stick models. The rotameric reorganization of Leu 2x46 is hindered in the inactive form by Asp 2x50 and Tyr 7x53 (shown in red)

Most GPCRs harbor a conserved binding site for sodium in the transmembrane bundle, close to the cytosolic side. Vickery et al^51^ nicely showed how the opening of a conserved hydrated channel in the activated state of the M_2_ muscarinic acetylcholine receptor allows the sodium ion to egress from this binding site into the cytosol. It was shown^9^ that this process contributes to the apparent binding energy of the agonist. Even though the role of the sodium ion seems clear, it was neither clear how the transfer of sodium to the cytosol is facilitated, nor how this is regulated. Figure 4 illustrates how Leu 2x46—the only highly conserved residue for which no important function has ever been proposed yet—plays a pivotal role in this process. In the inactive state (Figure 4(A)) Leu 2x46, Leu 3x43, and Ile 6x40 form a hydrophobic barrier that prevents the sodium ion from moving into the cytosol. We call the rotamer of Leu 2x46 in the inactive state the closed rotamer. In Figure 4(B), Leu 2x46 has been modeled in the open rotamer that is observed in the active state of the M_2_ receptor (Figure 4(C)). This opens a channel that is wide enough to let the sodium ion through.

Figure 4 shows that the open rotamer of Leu 2x46 cannot be reached in the inactive state because its space is occupied by the side chains of Asp 2x50 and Tyr 7x53; two residues often mentioned as pivotal in the activation process. A likely scenario is thus that the combined small motions that are facilitated by all the conserved weak spots and that are caused to work in unison by the agonist binding lead to just enough structural changes in the sodium surrounding to allow Leu 2x46 to move to the open rotamer. This is directly followed by the escape of the sodium, which in turn is associated by the larger rearrangements such as the big swing of Tyr 7x53 and the protonation of Asp 2x50.^51^ Figure 4 shows that there is ample space for an ion to escape from the sodium site to the cytosol. We find in most class A receptors a continuous cavity from just below Leu 2x46 to the cytosol. This cavity normally can accommodate the passage of a sodium ion.

### 3.6 The role of water molecules inside the helix bundle

The importance of water molecules for the function of GPCRs has been stated many times.^55^, ^56^, ^57^, ^58^ These articles classify water molecules in several ways. Venkatakrishnan et al used MD simulations and observed that (we cite) “the water molecules observed in GPCR crystal structures are not all equal: a few are stable in their crystallographically observed position, but most are highly mobile, spending only a small fraction of their time near that position”.^58^ They also observed a conserved group of waters at the location where the G protein will bind after agonist‐induced structural rearrangements have taken place. Fenalti et al^55^ stress the importance of waters in mediating interactions with the ligands, a fact that was also stressed by Koehl et al^56^ for the μ‐opioid receptor complexed with G_i_. Miller‐Gallacher et al^57^ studied the water molecules in a very stable variant of the β1‐adrenoceptor and conclude that the sodium ion and its (we cite) “associated water network stabilize the ligand‐free state of β1AR, but still permit the receptor to form the activated state which involves the collapse of the Na^+^ binding pocket on agonist binding”.^57^ Shalaeva et al^9^ note that there is a water‐filled tunnel from the sodium ion to the extracellular side of the receptor and speculate on the role of this tunnel as passageway for ions. We observe that in many GPCR structures adequate open space is available between Leu 2x46 and the cytosol for the sodium to escape. The activation‐associated motions at the cytosolic side of the helix bundle (Figure 2) further ensure that the sodium can reach the cytosol after Leu 2x46 adopted the open rotamer.

In summary, waters have shown to be an integral part of the GPCR structure, be essential for the function of the sodium ion, participate in the energetics of ligand binding, and mediate ligand interaction. The small local motions, that are all part of the activation process, change the local shape of the water‐filled pocket between the helices. Obviously, matter cannot be compressed nor can a vacuum come to exist, so these changes in the shape of the pocket necessarily lead to the displacement of atoms, and waters are perfectly suited to fulfill this task. Most small local motions center around irregularities in the helix backbone and thus must include the weakening of certain hydrogen bonds. Reorientation of water molecules seems the ideal mechanism to compensate for the associated loss in hydrogen bond energy.

### 3.7 The same helix weak spot can be induced by different sequence motifs

**FIGURE 5:**
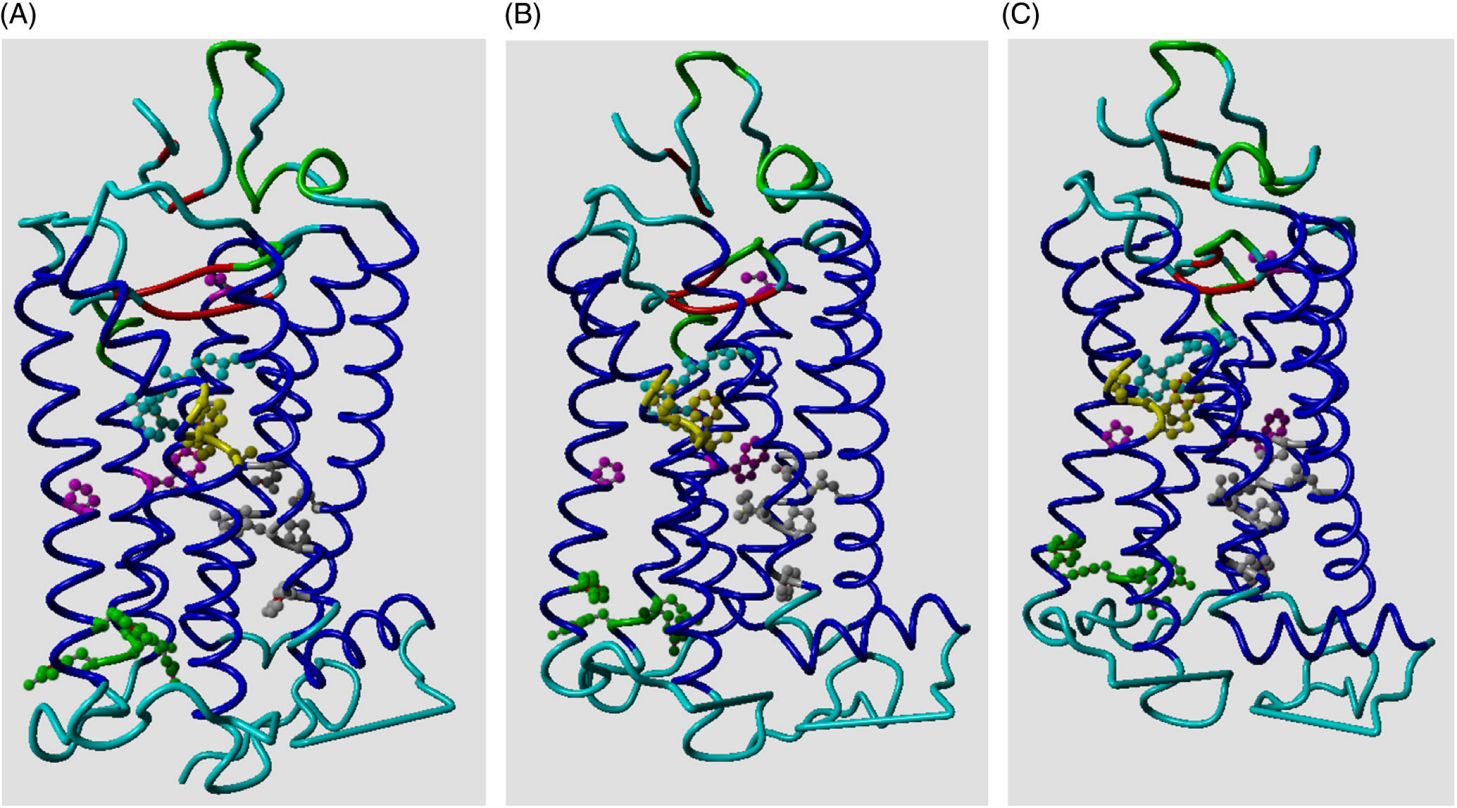
Location of conserved helix motifs in GPCRs. Bovine rhodopsin (PDB ID 1F88^4^) is shown to illustrate the location of conserved sequence motifs. To aid visual inspection of the location of residues, panels (A) and (C) are versions of panel B rotated around the vertical axis by approximately 15° clockwise and counter‐clockwise, respectively. Retinal is shown as a cyan ball‐and‐stick model for reference only. In the tube model helices are dark blue, strands are red, and the rest is cyan/green. Side chains of key, conserved residues are shown as ball‐and‐stick models that are colored by their most likely function. N1x50, L2x46, D2x50, N7x49, P7x50, and Y7x53 (in gray) all play a role in the sodium binding site, albeit that Y7x53 is also close to the G protein‐binding site. E3x49, R3x50, Y3x51, and Y5x58 (in green) are near the G protein‐binding site. C6x47, W6x48, and P6x50 (in yellow) are involved in ligand interaction. The functions of C3x25, W4x50, and P5x50 (in purple) are less clear. W4x50 is, in these views, in the back at the lipid surface of the helix bundle. C3x25 is, in these views, in the back of the molecule near the extracellular side, forming a cysteine bridge with the β‐hairpin that connects TM4 and TM5

Figure 5 indicates the location in the 7‐helix bundle of a series of conserved sequence motifs. The conserved residues in TM3 are not involved in helix irregularities while the conserved motifs in the other six helices all are responsible for weakening particular hydrogen bonds.^5^

**FIGURE 6:**
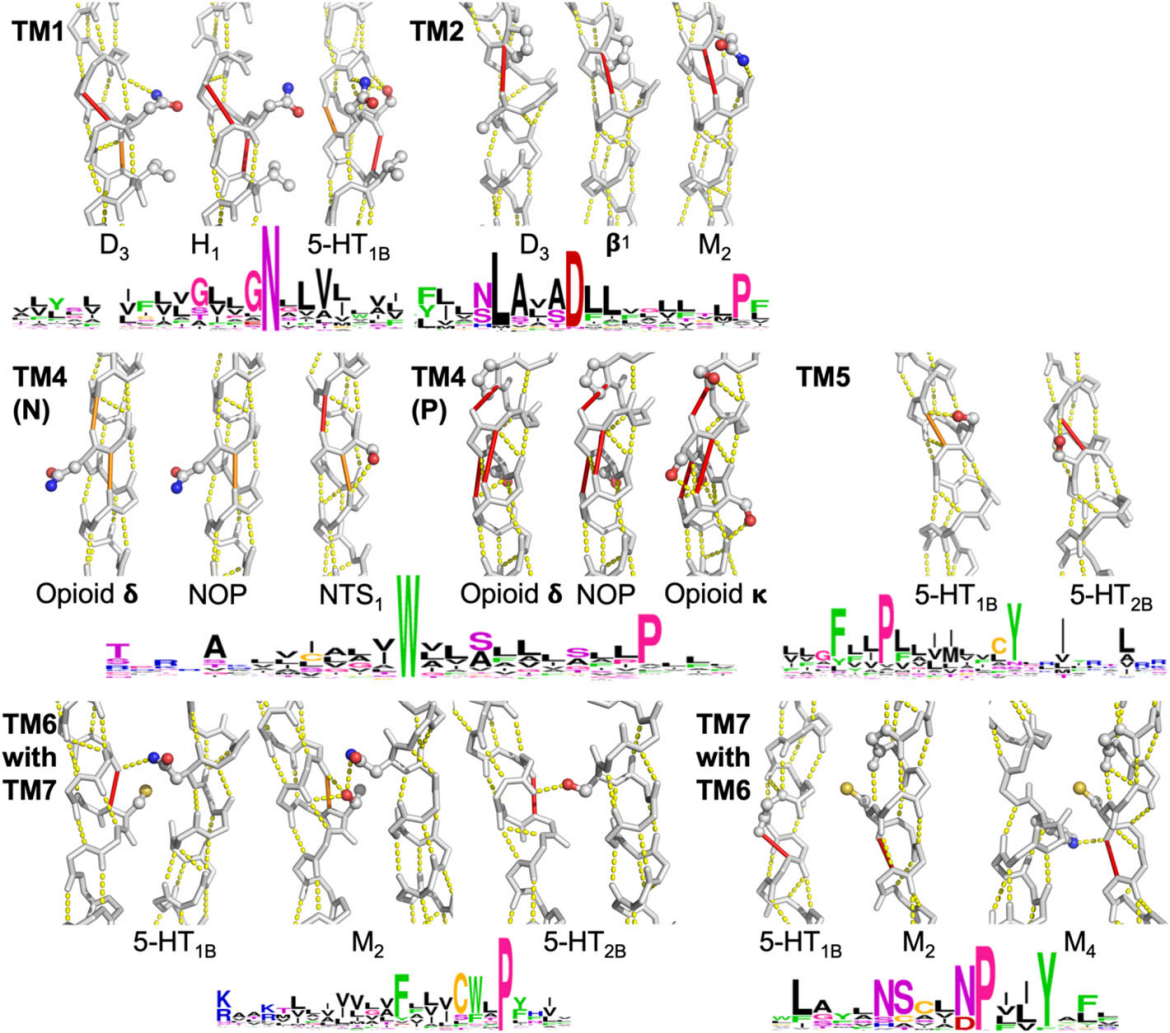
Examples of different sequence motifs causing similar helix hydrogen bond weakening in different GPCRs. For the six helices TM1, TM2, TM4, TM5, TM6, and TM7 examples of helix weakening are shown, with the sequence logo of the helix shown below the protein structures (colors in sequence logos are as in the GPCRdb^6^, ^7^). Dashed yellow lines indicate “normal” hydrogen bonds; solid orange lines indicate very weak hydrogen bonds; red lines connect hydrogen bond donor‐acceptor pairs that would form a hydrogen bond in a regular helix, but not in this particular GPCR helix. Side chains are shown in ball and stick view when relevant. Conserved weakened hydrogen bonds are indicated while non‐conserved missing hydrogen bonds are not shown. Receptors are indicated by their common name followed by the PDB identifier of the structure file: 5‐HT1B = serotonin receptor 1B, PDBid = 4IAR^59^; 5‐HT2B = serotonin receptor 2B, PDBid = 4IB4^60^; β1 = β_1_ adrenoceptor, PDBid = 4AMJ^61^; D3 = dopamine receptor 3, PDBid = 3PBL^8^; H1 = histamine receptor 1, PDBid = 3RZE^62^; M2 = muscarinic receptor 2, PDBid = 3UON^52^; M4 = muscarinic receptor 4, PDBid = 5DSG^63^; NOP = nociceptin receptor, PDBid = 5DHG^64^; NTS1 = neurotensin receptor 1, PDBid = 4XES^48^; Opioid δ = opioid δ receptor, PDBid = PDBid = 4N6H^55^; Opioid κ = opioid κ receptor, PDBid = 4DJH^65^

**FIGURE 7:**
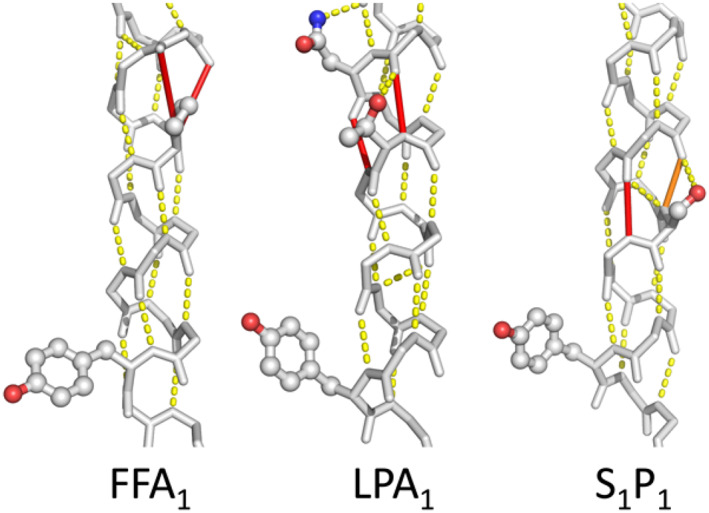
Motifs at different locations can support a similar motion. Receptors are indicated by their common name: FFA1 = free fatty acid receptor type 1, 4PHU^45^; LPA1 = lysophosphatidic acid receptor 1, 4Z35^66^; S1P1 = Sphingosine 1‐phosphate receptor 1, 3V2Y.^67^ Pro 5x50 (FFA1; left), Asn 5x47 and Thr 5x50 (LPA1; middle), and Ser 5x53 (S_1_P1; right) all cause an instability in the lipid receptors that can allow for similar helix reorientations at the cytosolic end of the helix including a similar reorientation of the tyrosine 5x58 at the cytosolic side of TM5

Figure 6 shows in each of the helices 1, 2, 4, 5, 6, and 7, at least one example of spatial conservation of helix weakening caused by PGDNST residues that are close to the weak spot. In a few cases, the location of the helix weakness is not exactly conserved but nevertheless supports the same rearrangement upon activation. In the lipid receptors, for example, we find a weakness in TM5 that is at a different location in each of the three structures studied (Figure 7). However, each of these weaknesses is located at the same face of the helix thereby allowing for a similar movement of the C‐terminal part of this helix.

In this paragraph we discuss the panels of Figure 6 systematically from top‐left (TM1) to bottom right (TM6 with TM7). **TM1**: The GNXXV motif in TM1 can also be SNXXV or GXNXXV. The two glycine residues allow for hydrogen bond weakening by facilitating local flexibility while the serine achieves this goal through a hydrogen bond with the local backbone. **TM2**: Aminergic receptors tend to have a proline at position 2x58. The muscarinic acetylcholine receptors lack this proline, but they nevertheless are weakened at the same position because of an asparagine at 2x58 that forms a hydrogen bond with the local backbone. Position 2x54 is a glycine in many aminergic receptors and in all muscarinic receptors. **TM4 N**: Asparagine 4x46 interacts with TM2 and this pulls at the local backbone in the NOP‐ and opioid δ receptors. Serine 4x47 makes a hydrogen bond with the backbone that destabilizes TM4 of the NTS_1_ receptor in the same way, despite asparagine 4x46 being replaced by an isoleucine in this receptor. **TM4 P**: Opioid receptors have a proline at 4x59, normally combined with a serine at position 4x54. The kappa opioid receptor achieves a highly similar helix‐weakening pattern through hydrogen bonds with the local backbone by serine residues at the positions 4x53, 4x54, 4x55, and 4x59. **TM5**: Serine 5x43 hydrogen bonds to the local backbone in TM5 of the 5‐HT_1B_ receptor. This weakens the same hydrogen bond as the one weakened by the combination of a glycine at the same position (5x43) and a serine at position 5x44 in the 5‐HT_2B_ receptor. **TM6** with **TM7**: The cysteine 6x47 of the CWXP motif in helix 6 forms a hydrogen bond with the local backbone (left panel). Such hydrogen bonds are generally not very strong, but in this case the cysteine is “helped” by the side chain NH_2_ group of asparagine 7x45. Muscarinic receptors have a threonine at position 6x47 that can form this same hydrogen bond in a more normal way. The 5‐HT_2_ receptors have a methionine at position 6x47. In these receptors a hydrogen bond is formed between TM7 (serine 7x45) and the carbonyl of residue 6x44. **TM7** with **TM6**: TM7 of most aminergic receptors is weakened at the cytosolic side of glycine 7x41. Surprisingly, a cysteine at this position can create the same instability in the muscarinic acetylcholine receptors when “helped” by a glycine at position 7x37. The cysteine distorts the helix because it is pushed into the helix backbone by the highly conserved tryptophan 6x48 of TM6. This is shown in the muscarinic receptor 4.

In some cases the residues that combine into the mobility‐supporting sequence motif are not located at the same position in the helix. Figure 7 shows examples in three receptors.

## 4 CONCLUSIONS

Residues that are important for a protein's function tend to remain conserved during evolution, with residues important for the primary function remaining most conserved. Residues associated with auxiliary functions may vary over evolutionary time between sub‐families.^68^ This idea was worked out in great detail for the GPCR family by Oliveira et al^69^, ^70^, ^71^ who introduced correlated mutation analysis (CMA) to find those residue positions that are conserved within sub‐families, but differ between them. Kuipers et al^72^ showed that CMA can also be used to understand the role of external factors, such as the optimal wavelength of a photon, the binding mode of an endogenous peptide ligand, or the binding of exogenous small molecule ligands. We show that small structure changes that are important for GPCR activation can be achieved in nearly identical ways by different combinations of residues.

It has often been stated that structures are more conserved than sequences, and it is also true that the location of functional residues is even more conserved.^73^, ^74^ We now see for the first time that there is a yet higher level of conservation: activation facilitated by plasticity in the structure caused by the absence of hydrogen bonds. The implications of this new level of conservation for study of the evolution of GPCRs are enormous, but beyond the scope of this article. We believe, however, that the conservation of something that is not there (after all, it is the *absence* of hydrogen bonds that is conserved) provides radically new input into the study of this very large protein family. It is likely that similar effects play a role in many protein families in which structure deformation is part of the activation process, which would make our finding a novel concept in the study of protein families in general.

We hope that this study will help in the design of new GPCR stabilization strategies so that many more GPCRs can be crystallized and have their coordinates determined so that they can contribute to research in which GPCR structures play a crucial role, especially in the area of drug design. We also hope that it will help make structures available with different combinations of unmutated key residues. These will be important for deepening our understanding of the GPCR (in‐)activation process, which in turn will be crucial for the design of medicines that can be better tailored to the intended biological effect, be more specific, and have fewer side effects.

### PEER REVIEW

The peer review history for this article is available at https://publons.com/publon/10.1002/prot.26179.

## Supporting information


**Appendix** **S1**: Supporting Information.

## Data Availability

Any relevant data is available at swift.cmbi.umcn.nl/gv/GPCR/.
